# Phosphorylation of Large T Antigen Regulates Merkel Cell Polyomavirus Replication

**DOI:** 10.3390/cancers6031464

**Published:** 2014-07-08

**Authors:** Jason Diaz, Xin Wang, Sabrina H. Tsang, Jing Jiao, Jianxin You

**Affiliations:** 1 Department of Microbiology, University of Pennsylvania Perelman School of Medicine, Philadelphia, PA 19104, USA; E-Mails: jdia@mail.med.upenn.edu (J.D.); wangxin2@mail.med.upenn.edu (X.W.); sathw323@mail.med.upenn.edu (S.H.T.); 2 Department of Pathology and Laboratory Medicine, Children’s Hospital of Philadelphia, Philadelphia, PA 19104, USA; E-Mail: jiaoj1@email.chop.edu

**Keywords:** MCPyV, Large T antigen, polyomavirus replication, phosphorylation

## Abstract

Merkel Cell Polyomavirus (MCPyV) was recently discovered as a novel human polyomavirus that is associated with ~80% of Merkel Cell Carcinomas. The Large Tumor antigen (LT) is an early viral protein which has a variety of functions, including manipulation of the cell cycle and initiating viral DNA replication. Phosphorylation plays a critical regulatory role for polyomavirus LT proteins, but no investigation of MCPyV LT phosphorylation has been performed to date. In this report mass spectrometry analysis reveals three unique phosphorylation sites: T271, T297 and T299. *In vivo* replication assays confirm that phosphorylation of T271 does not play a role in viral replication, while modification at T297 and T299 have dramatic and opposing effects on LT’s ability to initiate replication from the viral origin. We test these mutants for their ability to bind, unwind, and act as a functional helicase at the viral origin. These studies provide a framework for understanding how phosphorylation of LT may dynamically regulate viral replication. Although the natural host cell of MCPyV has not yet been established, this work provides a foundation for understanding how LT activity is regulated and provides tools for better exploring this regulation in both natural host cells and Merkel cells.

## 1. Introduction

Merkel Cell Polyomavirus (MCPyV) is the first human polyomavirus to be linked to a human cancer, Merkel Cell Carcinoma (MCC) [1,2,3]. Since its discovery in 2008, this virus has been found clonally integrated in a majority of MCC tumors. In most MCPyV-related MCC tumors, the major viral protein, Large T antigen (LT), has been mutated such that it is expressed in a truncated form [4]. These tumor-derived truncated LT proteins retain their binding sites for retinoblastoma protein (pRb) and DnaJ family heatshock proteins, thereby driving proliferation [1,4]. Indeed, at least one study has shown that tumors with knocked-down LT protein regress rapidly in a xenograft model [5]. Both LT and the splice variant, small T antigen (sT) have been shown to have oncogenic properties [4,5,6,7], although the precise contribution of each protein to the process of tumorigenesis is still unclear.

MCPyV appears to be a natural resident of the skin microflora, and is acquired early in life [8,9,10]. While considerable efforts have been made to better understand MCPyV’s oncogenic potential, especially in MCC tumors, comparatively little work has been done to better understand this virus’s basic life cycle. The lack of a relevant cell culture system for propagating virus has made investigations of its basic virology difficult; however, ectopic expression of MCPyV LT in cell lines such as HEK 293, C33A and U2OS has been valuable in enhancing our understanding of MCPyV’s interaction with host cells [11,12,13,14].

Our lab has previously characterized MCPyV LT’s interaction with the host cell to stimulate replication [14]. In that study we showed by immunoflourescent staining (IF), fluorescent *in situ* hybridization (FISH) and BrdU staining that MCPyV LT proteins form large nuclear foci which contain actively replicating plasmids carrying the viral origin of replication (Ori). We also showed that several cellular factors colocalize to these foci, including: the double bromodomain protein, Brd4, the PCNA loading protein replication factor 1 (RFC1), and the single-stranded DNA binding protein RPA70. In another study we demonstrated that full length MCPyV LT activates host DNA damage response (DDR) pathways and dramatically alters the host cell cycle [12]. Additionally, members of the DDR pathway were seen to colocalize with nuclear foci containing actively replicating viral genomes, potentially contributing to viral replication [13]. While it is still unclear whether DDR activation and recruitment upon LT expression is a side-effect of active viral replication and/or LT helicase activity, or if this activation is being actively subverted and manipulated by MCPyV, the link between MCPyV LT expression and DDR activation is well established. This DDR activity, coupled with LT’s ability to dramatically alter the host cell cycle, may provide enough low-level genomic instability to lead to integration of its genome into the host cell genome, which appears to occur in the majority of MCPyV-related MCC tumors studied to date.

Merkel cells may not represent the natural host cell of MCPyV and may pre-dispose MCPyV to randomly integrate its genome. Indeed, the prototypical polyomavirus, Simian Virus 40 (SV40) has a transforming phenotype in cell lines that are non-permissive for viral replication [15]; Merkel cells may similarly represent a non-permissive host for MCPyV. A better understanding of how MCPyV replication is regulated would provide a clearer framework for understanding how infection may be altered in Merkel cells and lead to integration of the mutated viral genome.

SV40 LT has been a model for understanding eukaryotic replication for decades [16]. SV40 LT is recruited to the viral Ori through its origin binding domain (OBD), which recognizes GAGGC pentanucleotide repeats arranged symmetrically within the Ori. LT then oligomerizes into two hexameric protein complexes arranged in a head-to-head fashion. The C-terminal helicase domains make non-specific contacts with an extended palindrome and an A/T rich tract flanking the central pentanucleotide repeats; these become the initial sites of unwinding. LT then acts as a helicase to unwind the viral genome and recruits cellular factors to begin replication [16,17].

Phosphorylation has been a well-established mechanism by which SV40 LT replication is regulated [18]. T124 was identified as a critical residue for regulating SV40 LT-mediated viral replication; removal of this phosphorylation either biochemically or genetically abrogated replication [19,20,21,22]. Intensive biochemical studies demonstrated that this phosphorylation plays an important role in mediating interactions between both hexamers at the Ori. Alanine mutants are defective in forming double-hexamer complexes and unwinding the viral origin [23,24].

Somewhat paradoxically, early biochemical analyses of purified SV40 LT seemed to indicate that phosphatase treatment could actually stimulate viral replication [25,26]. It was later clarified that, in addition to phosphorylation at T124, there are serine phosphorylation modifications nearby which have an inhibitory effect on viral replication [27]. These phosphorylation events seem to accumulate throughout the course of infection [28]. These observations led to a model where T124 phosphorylation stimulates replication, while subsequent phosphorylation at neighboring serines dampen this effect, potentially altering LT’s activity on the viral genome to favor transcription of the capsid genes [18].

No such analysis of MCPyV LT phosphorylation has yet been reported. We sought to provide an initial framework for understanding the regulation of MCPyV LT’s functions by performing a proteomic analysis to search for relevant phosphorylation sites. Our studies identify three phosphorylation marks on MCPyV LT; T271, T297 and T299. We found that T271 had no effect on replication, while T297 and T299 phosphorylation had antagonistic effects. Both T297 and T299 altered the binding affinity of MCPyV for the viral Ori while leaving unwinding and helicase functions largely intact. Taken together, our data reveal a dynamic interplay between multiple phosphorylation sites, which together regulate LT’s ability to initiate replication at the viral origin.

## 2. Results

### 2.1. Mass Spectrometry Identifies T271, T297 and T299 as Phosphorylation Sites on MCPyV LT

Polyomavirus LT proteins perform a large variety of functions in infected cells to establish a replicative niche; these functions include manipulation of the host cell cycle through its DnaJ domain and pRb binding domain, regulation of viral transcription, and initiation of viral genome replication by acting as the viral helicase [3,18,29]. Phosphorylation of LT has been well established as a mechanism of regulating its function, especially as a replication initiator protein.

**Figure 1:**
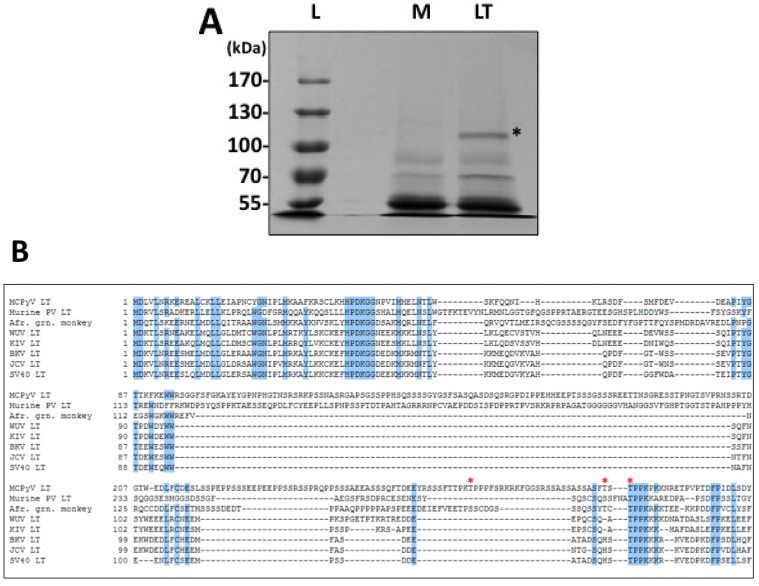
Identification of MCPyV LT phosphorylation sites. (**A**) Affinity-tagged MCPyV LT was transfected into HEK 293 cells. Forty-eight hours post transfection, lysates from transfected (LT) or untransfected (Mock, M) cells were purified with IgG-Sepharose beads. Bound proteins were cleaved from beads with TEV protease, separated by SDS-PAGE and stained with Coommassie brilliant blue. The band corresponding to MCPyV LT (*) was excised and analyzed by mass spectrometry. L—Protein Marker ladder; (**B**) Alignment of the N-terminal portion of various polyomavirus LT proteins. The three phosphorylated threonines identified by the proteomic analysis in (A) are indicated by the red asterisks (*). Conserved residues are highlighted in blue (80% conservation).

To date, no analysis of MCPyV LT phosphorylation has been performed. To get a broad view of MCPyV LT phosphorylation, we performed a mass spectrometry analysis to identify potential LT phosphorylation sites. MCPyV LT was first affinity tagged with two IgG binding domains from *S. aureus* Protein A and a Tobacco Etch Virus (TEV) Protease cleavage site. This construct was then ectopically expressed in HEK 293 cells. The protein was affinity purified on IgG-Sepharose beads, separated by SDS-PAGE and stained with Coommassie blue (Figure 1A). The visible band corresponding to MCPyV LT was excised and analyzed by mass spectrometry.

Trypsin-digested peptides from LT were purified and analyzed by LC/MS/MS. The peptides covered 45% of MCPyV LT and identified two unique peptides with a shift of 80 Daltons over the predicted size, indicating a potential phosphorylation modification at T271 and T299. We repeated this analysis using a titanium oxide column to enrich for negatively charged peptides, such as those with phosphorylated residues. The peptides from this purification covered 25% of MCPyV LT and identified potential phosphorylation at T271 and T297 (Supplementary Table S1).

To better understand how these phosphorylation modifications may regulate MCPyV LT function, we aligned the amino acid sequence of various representative polyomavirus LT proteins (Figure 1B). T271 localized to the serine rich unique region of MCPyV LT spanning amino acids 95–290. Of the polyomavirus LT proteins analyzed, only murine polyomavirus contains such a tract; however, it is not well conserved with MCPyV LT. Although the lack of homology prevented us from making functional predictions of T271 phosphorylation, it is interesting to note this threonine was identified by multiple peptides in both mass spectrometry purification schemes (Supplementary Table S1), giving us high confidence that this site is phosphorylated when LT is expressed in cells.

**Figure 2:**
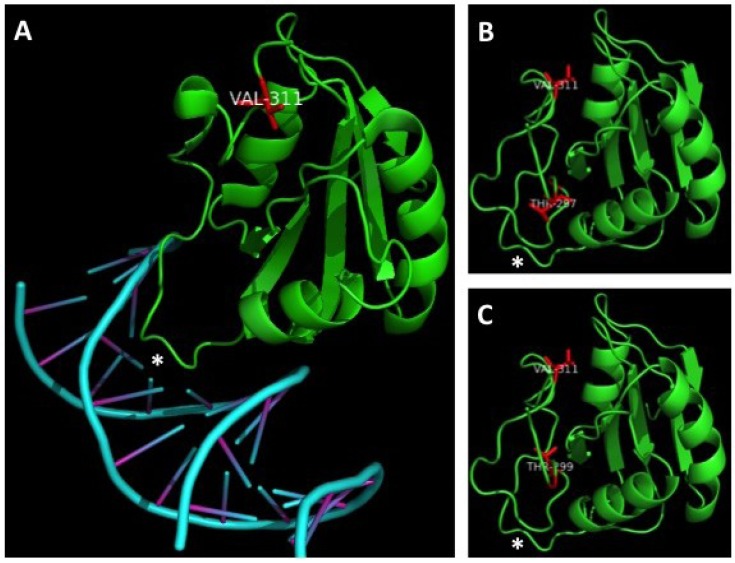
Modeling MCPyV LT’s interaction with DNA. (**A**) Phyre2 and PyMOL software was used to model the MCPyV LT protein origin binding domain (OBD) contacting DNA as reported by Harrison and colleagues [30]. The asterisks indicate the loop of the OBD that makes contacts with DNA; (**B**) The structure modeled in (**A**) was extended to aa 290 and modeled using Phyre2 and PyMOLsoftware. The model was rotated to match the orientation of the structure in (**A**). T297 is highlighted in red and appears to face—and possibly contact—DNA; (**C**) The same model in (**B**) was labeled to show T299, which appears to face away from DNA, possibly to interact with adjacent LT monomers or hexamers. Valine 311 is labeled in all three structures to aid in comparison.

Threonine 297 is not well conserved amongst polyomavirus LT proteins. Its function was not readily apparent to us based on homology, although its close proximity to the OBD led us to predict that it might have some impact on viral replication. Modeling of the OBD (aas 308–433) using the structure published by Harrison and colleagues (Figure 2A) [30], and extending their structure to include this threonine supported this hypothesis; T297 appears to face the protein/DNA interface and might even make direct contacts with DNA (Figure 2B). In contrast to the two threonines discussed above, T299 is a highly conserved site found in all polyomavirus LT proteins analyzed. The homologous site in SV40 LT, T124, has been well established as a key regulator of LT mediated DNA replication. Phosphorylation of this threonine in SV40 is thought to regulate double-hexamer interactions on the viral origin to stimulate unwinding and melting of the DNA. Alanine substitutions of this site in SV40 LT completely abolish LT-mediated DNA replication. Our modeling of the OBD showed that this threonine does not directly face the protein/DNA binding interface and likely plays a role in protein-protein interactions between LT monomers and/or hexamers (Figure 2C). The crystal structure of the MCPyV OBD was solved in complex with DNA; we cannot rule out the possibility that T299 may interact with DNA in steps prior to hexamer assembly on the origin.

The sequence analysis and modeling led us to believe that phosphorylation of at least a subset of the threonines identified would play a role in viral DNA replication. Our lab has previously studied MCPyV LT-mediated replication in C33A cells, an HPV-negative cervical cancer cell line; we therefore took advantage of this system to probe the potential roles of these threonines in viral DNA replication.

### 2.2. MCPyV LT Phosphorylation Affects the Formation of Viral Replication Centers

To begin elucidating the role(s) these phospho-sites might play in replication, we generated alanine point mutants at each threonine identified by the mass spectrometry analysis. We then co-transfected these constructs with a plasmid containing the MCPyV Ori into C33A cells and stained these cells for LT and various replication factors, as has been described in our previous study [14].

We assessed these mutants for their ability to form viral replication centers, which appear as nuclear foci, in transfected nuclei by immunoflourescence (IF). We also assessed their ability to recruit factors known to co-localize with MCPyV replication foci, including Brd4, RFC1 and RPA70.

**Figure 3:**
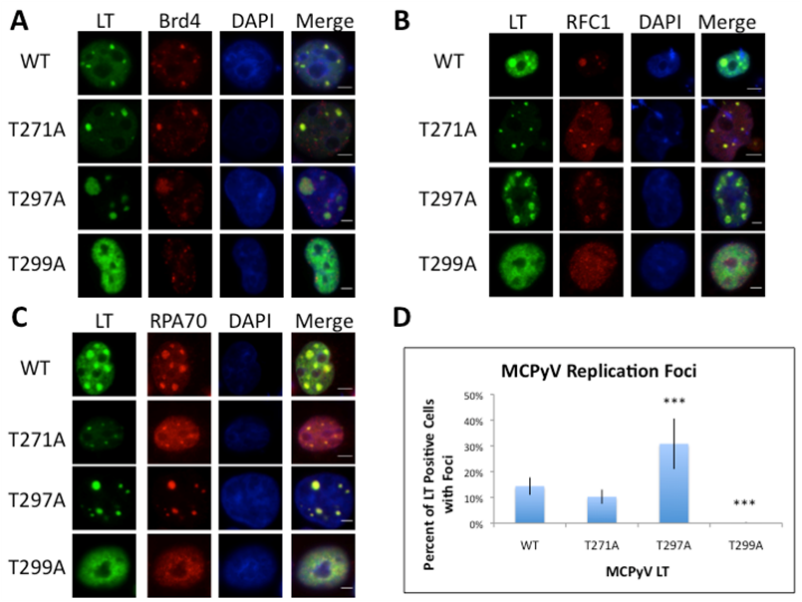
MCPyV LT phospho-mutant proteins form viral replicaiton foci at altered efficiencies. (**A**–**C**) C33A cells were co-transfected with an MCPyV Ori plasmid and the indicated MCPyV LT phospho-mutant. Forty-eight hours post transfection, cells were fixed and stained for LT (green) and the indicated cellular factor (Red). Nuclei were counterstained with DAPI. Bar = 3 µm; (**D**) Nuclei stained positively for LT as shown in (**A**–**C**) were scored for the presence of viral replication foci (at least 150 LT positive nuclei were counted in triplicate per transfection). Bar indicates standard deviation from the mean from at least three independent experiments. Statistical significance was calculated against WT LT using a one-way ANOVA (*** *p* < 0.0001).

As has been shown previously, wild-type (WT) LT formed punctate foci in nuclei when co-transfected with a construct containing the MCPyV Ori. Our previous studies have confirmed that under these transfection conditions these foci contain the MCPyV Ori plasmid (as shown by FISH) and are actively replicating (assessed by incorporation of BrdU) [13]. Both the T271A and T297A mutants formed replication foci while T299A completely failed to assemble replication foci (Figure 3A–C). The T271A and T297A mutant LT proteins formed replication foci at altered rates. Compared to WT LT, which formed replication foci in about 15% of LT positive nuclei, the T271A mutant had a small decrease in the frequency of replication focus formation (about 10%) (Figure 3D). This difference, however, was not statistically significant in a one-way ANOVA test. In contrast, the T297A mutant exhibited twice as many LT-positive nuclei with replication foci. For constructs which formed replication foci, recruitment of cellular factors did not seem to be affected; WT, T271A and T297A LT proteins all recruited Brd4, RFC1 and RPA70 proteins at similar rates. Colocalization with T299A, which did not form replication foci, was not evident (Figure 3 and Supplementary Figure S1). As has been seen previously, replication foci came in a variety of sizes, and nuclear swelling was often evident, especially in cells expressing T297A LT, possibly to accommodate rapidly replicating plasmids.

### 2.3. MCPyV LT Phospho-Mutant Proteins Have Altered Replication Capacities

**Figure 4:**
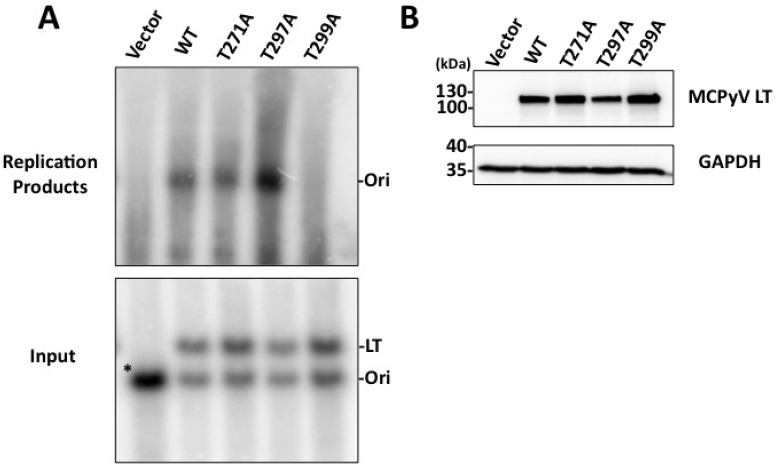
MCPyV LT phospho-mutant proteins replicate plasmids containing the viral Ori to differing degrees. C33A cells were co-transfected with a MCPyV Ori plasmid and the indicated MCPyV phospho-mutant LT. Forty-eight hours post transfection cells were split and extracted for total cellular DNA or total proteins. (**A**) Southern blotting of whole genomic DNA. Both the MCPyV Ori and MCPyV LT plasmids use the same vector backbone and are both recognized by the Southern blot probe. 15 µg of DNA was digested with BamHI and DpnI to detect replicated origin plasmid (Replicated Products, top); replicated Ori plasmid is indicated. 2 µg of DNA was digested with only BamHI to show equal loading (Input, bottom); Ori and LT plasmids are indicated. The vector control plasmid for LT is almost identical in size to the Ori plasmid, causing both plasmids to co-migrate in the blot (asterisk (*), first lane, bottom panel); (**B**) Total protein extracts were western blotted to detect MCPyV LT and GAPDH. Southern and western blots are representative of at least three experiments.

Our immunoflourescent studies indicated that at least a subset of the threonines identified in our proteomic analysis affect viral genome replication. To more rigorously examine these mutants’ ability to replicate plasmids containing the viral Ori, we performed Southern blotting experiments to detect replicated plasmids. C33A cells were co-transfected with a MCPyV LT construct and a plasmid containing the viral Ori. Two days after transfection, cells were harvested and divided into two fractions. Whole genomic DNA extracts were prepared from one fraction while proteins were collected from the other fraction. Whole genomic DNA was then digested with BamHI to linearize the plasmids. The DNA was then detected with ^32^P-labeled MCPyV Ori plasmid as a probe. The LT expression construct is made from the same vector backbone as the MCPyV Ori plasmid, and is therefore detected as a second, higher molecular weight band in our Southern blot. Additionally, the vector control for MCPyV LT is almost identical in size to the MCPyV Ori plasmid, so these two plasmids co-migrate as one band in our blots (Figure 4A, bottom). Whole genomic DNA was also digested with DpnI to reveal newly replicated DNA (Figure 4A, top).

The Southern blotting results complemented what was seen by immunoflourescence (Figure 3). Compared to WT MCPyV LT, the T271A mutant replicated the MCPyV Ori plasmid almost as well. In contrast, the T297A mutant, which had twice as many nuclei with replication foci as WT (Figure 3D), had a robust replication phenotype that was well over that seen for WT.

This was especially striking given that both the amount of MCPyV Ori input plasmid and the protein level of T297A LT was less than WT (Figure 4). Finally, the T299A mutant, which failed to form replication foci as seen by IF, was unable to replicate Ori plasmids at a level detectable by Southern blot (compare Figure 4A, top panel, Vector and T299A lanes). This agrees with what was seen in SV40, where the homologous mutant, T124A, failed to replicate viral genomes.

### 2.4. T297A and T299A Phospho-Mutant LT Proteins Bind the Viral Ori with Altered Affinity

Our studies up to this point confirmed that the T297A and T299A mutants had greatly altered replication phenotypes; T271A by contrast showed a modest effect on replication. To get a firmer understanding of the molecular basis for the replication phenotypes we observed, we next sought to examine the binding, unwinding and helicase activities of these mutants. We focused our analyses on the T297A and T299A mutants, which had dramatic replication phenotypes.

**Figure 5:**
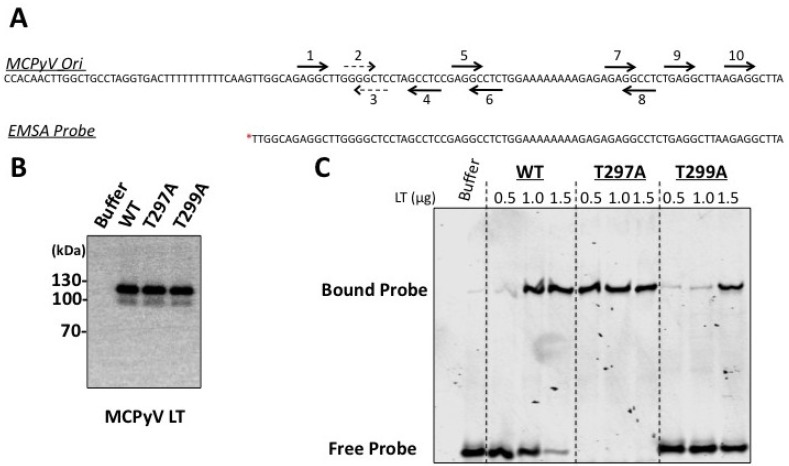
MCPyV LT phospho-mutants bind the viral Ori with different affinities. (**A**) Schematic of the MCPyV Ori and the EMSA Probe. Only one strand of DNA is shown for clarity. The MCPyV Ori sequence was cloned from the R17a isolate of MCPyV into a pcDNA4c vector [14]. This origin was used for replication assays (Figure 3 and Figure 4). Consensus GAGGC pentanucleotide repeats which are recognized by the OBD of LT are marked with arrows and numbered as was reported by Kwun *et al.* [31]. Arrows with dashed lines indicate imperfect pentanucleotides. The EMSA Probe was generated by PCR amplification of the indicated region of the MCPyV Ori. This PCR product was 5' end-labeled with [^32^P-γ] ATP using T4 polynucleotide kinase (indicated by red asterisk); (**B**) Western blot of purified MCPyV proteins (0.25 µg) used in EMSA. The buffer control contained residual TEV protease (also in LT samples); (**C**) Electromobility shift assays were performed with the EMSA probe in (**A**) and increasing amounts of MCPyV wild type or phospho-mutant LT affinity purified from HEK 293 cells. Reactions with buffer and residual TEV protease served as a negative control (first lane). Positions of free probe and LT bound probe are indicated. Data in (**B**,**C**) are representative of at least three experiments.

Polyomavirus LT binding of the viral Ori is mediated by its OBD, which recognizes GAGGC pentanucleotide repeats in the Ori. The MCPyV Ori more closely resembles that of murine polyomavirus; it contains eight perfect GAGGC pentanucleotides and two imperfect pentanucleotides, with two A/T rich regions interspersed between these repeats (Figure 5A) [30,31]. Previous studies have shown that pentanucleotides 1, 2, 4 and 7 are essential for replication [31].

To test origin binding, we performed electromobility shift assays (EMSA). Affinity tagged MCPyV LT constructs were transfected into HEK 293 cells. Forty-eight hours later, LT proteins were immunopurified and cleaved with TEV protease (Figure 5B). Various amounts of purified proteins were then incubated with a ^32^P-labeled PCR product encompassing all ten pentanucleotides and one of the two A/T tracts (Figure 5A, EMSA probe). Previous work with the SV40 Ori demonstrated that using Ori probes that lacked either the A/T rich tract or early palindrome region of the Ori revealed a defect in double hexamer formation for T124A mutants [23]. We predicted that the T299A mutant would exhibit a similar double hexamer defect in similarly altered Ori probes; however, given that one of the key pentanucleotides required for MCPyV replication (#7) lies outside one of the two A/T rich tracts of the MCPyV Ori, it was impossible to directly copy the unique architecture of the artificial probes generated in that study [23,30,31]. Following these observations, we chose to omit one A/T tract but retain all ten pentanucleotide repeats in the hopes of seeing this phenotype. In addition, the ATP analogue AMP-PNP was included to stimulate hexamer formation but inhibit ATPase activity, which would unwind the double-stranded probe [32]. Protein/DNA complexes were resolved on a non-denaturing polyacrylamide gel in TBE.

All mutant MCPyV LT proteins were able to bind the probe, albeit with different affinities. The WT LT showed robust binding beginning at 1 µg purified protein; probe binding was further enhanced when more LT was added (Figure 5C). In contrast, the T297A mutant achieved maximal probe binding at 0.5 µg, indicating a more robust affinity for this probe than WT. This phenotype agrees with the previous observation that this mutant replicates plasmids with the MCPyV Ori to a high degree (Figure 3 and Figure 4). On the other hand, the T299A mutant exhibited an attenuated affinity for the EMSA probe; only at the highest dose (1.5 µg) was binding evident, and still not to the level of either WT or T297A LT proteins. Our EMSA studies with T299A did not reveal single hexamers, which would migrate faster than the double hexamers binding our probe. It is possible that T299A does not have a double hexamer defect like its SV40 homologue, or that the probe used in our study was not sufficiently small to reveal such a phenotype. Native PAGE analyses of these proteins was unable to resolve this question (data not shown). The technical limitations of our assay therefore preclude making any statements about the ability of these mutants to form single or double hexameric complexes. The attenuated binding phenotype, however, agrees with the observation that this mutant fails to replicate plasmids containing the viral Ori (Figure 3 and Figure 4) and agrees with what has been reported for SV40 LT T124A binding [33].

### 2.5. MCPyV Phospho-Mutant LT Proteins Exhibit Similar Unwinding and Helicase Activities

**Figure 6:**
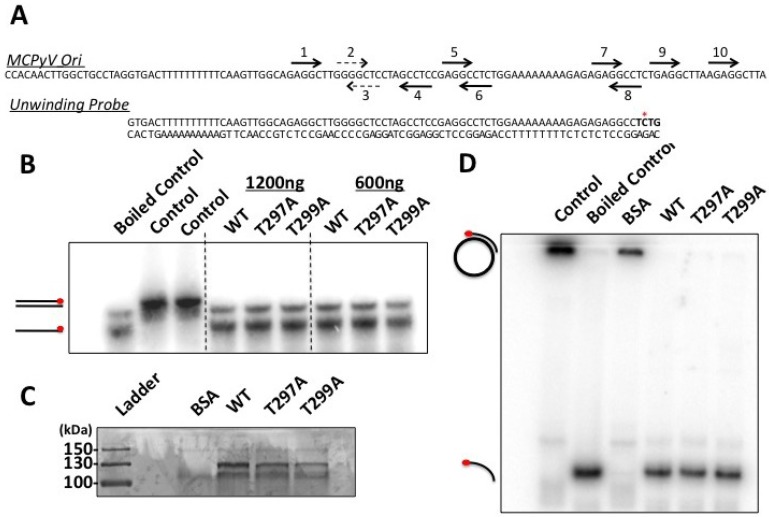
MCPyV LT phospho-mutants have similar unwinding and helicase activities. (**A**) Schematic of the Unwinding Probe. The Unwinding Probe was generated by annealing two complimentary oligonucleotides spanning the indicated sequence. The duplexed oligo contains a four-nucleotide overhang that was filled in by Klenow and [^32^P-α] dCTP so that only one strand was labeled. The filled-in nucleotides are marked in bold, and the radiolabeled dCTP is indicated by an asterisk. The MCPyV Ori sequence depicted in Figure 5A is shown for reference; (**B**) Unwinding assays were performed with varying amounts of affinity purified MCPyV LT (wild-type or phospho-mutant). Samples without purified protein served as a negative control. One sample was boiled to show the migration of unbound probe. Data are representative of three independent experiments; (**C**,**D**) Constructs expressing affinity tagged MCPyV wild-type or phospho-mutant LT were transfected into 293 cells. Proteins were harvested 48 h post-transfection and LT was immunopurified on IgG-conjugated beads. Half of the beads with bound LT were boiled in sample buffer and resolved on an SDS-PAGE followed by Coommassie staining (**C**) while the remaining beads were used in the helicase assay (**D**). Beads incubated with 1% bovine serum albumin served as a negative control (BSA). Helicase reaction mix incubated at room temperature (Control) or at 95 °C for 5 min (Boiled Control) served as controls for partially duplex and unwound substrate, respectively. Data shown are representative of at least three independent experiments.

After initial binding of the viral Ori, polyomavirus LT proteins form two hexameric complexes that then untwist and unwind the origin DNA to form an initial bubble of single-stranded DNA. To test these mutants for their ability to unwind a double-stranded Ori sequence, we generated a probe that contained both A/T tracts and the first eight pentanucleotide repeats (Figure 6A, Unwinding Probe). This probe was generated by annealing two oligonucleotides which form a duplex with a four nucleotide overhang; this overhang was filled in with the Klenow fragment of DNA Polymerase and ^32^P-labeled dCTP, generating a double stranded probe with one labeled strand. This probe was then incubated with purified MCPyV LT in conditions that promote ATPase activity. Reactions were then stopped with the addition of SDS and EDTA, and the DNA was resolved on a non-denaturing polyacrylamide gel. All LT constructs were able to unwind the probe (Figure 6B). This result was helicase dependent, because an MCPyV LT mutant, E627A, that we have previously described failed to unwind our probe (Supplementary Figure S2) [12]. The T297A mutant, which showed a high affinity for the Ori (Figure 5C) showed unwinding activity similar to WT LT; because WT LT’s activity was close to maximal unwinding in this assay (compare Boiled Control lane with WT LT lanes, Figure 6B) we are unable to say whether this mutant might have increased unwinding activity over WT activity.

We also tested whether MCPyV LT could unwind a mutated Ori sequence. We introduced the Ori350 point mutation to pentanucleotide 7 in our assay; this mutation was reported by our lab and others to have partially abrogated replication due to reduced binding of LT to the origin (Supplementary Figure S2) [13,31]. LT was still able to unwind this mutant sequence; this is likely because either the amount of LT protein used in our *in vitro* settings, or the extended reaction time (1 h) compared to the EMSA (20min) can compensate for the reduced binding of LT to this mutant Ori.

Following unwinding of the viral Ori, hexameric LT complexes then function as a DNA helicase that translocates along DNA to separate double stranded DNA. To test this function, we employed a helicase assay that has been previously reported from our lab [12]. This assay uses a circular, partially duplex DNA substrate as a probe for LT helicase activity. The probe does not contain MCPyV Ori sequences; DNA binding is mediated by non-specific interactions in the helicase domain. The reaction is carried out with affinity purified LT proteins still immobilized to the affinity resin. After washing bound proteins, half of the resin with bound LT protein was boiled in sample buffer and western blotted to detect protein levels (Figure 6C). The other half of the resin was incubated with the ^32^P labeled probe in reaction buffer. Helicase activity separates the labeled oligonucleotide from the circular template, allowing the probe to migrate faster during electrophoresis.

Our results show that the mutant MCPyV LT proteins had similar helicase activity as WT LT (Figure 6D). In the conditions used, WT LT was able to maximally unwind the labeled probe; therefore we cannot conclude whether either of the mutants had enhanced helicase activity from this assay. Interestingly, the T299A mutant, which failed to replicate the viral origin (Figure 4) did not have attenuated helicase activity in this experiment. This has been previously reported for the T299A homologue in SV40 LT, indicating that this mutant’s helicase functions remain intact, and the block to replication is primarily due to an inability to bind the origin efficiently (Figure 5C) [20]. In contrast, the T297A mutant likely replicates to a high degree solely due to an increased affinity for the origin, as its unwinding and helicase activities were not markedly different from WT LT. Taken together, our data indicate that T297 phosphorylation plays a direct role in binding of the origin, while T299 phosphorylation affects both origin recognition and possibly initial unwinding of the origin DNA, similar to its function in SV40 LT.

## 3. Experimental

### 3.1. Cell Lines, Cell Culture and Transfection

HEK 293 and C33A cell lines were maintained in Dulbecco’s Modified Eagle Medium (DMEM; Invitrogen, Grand Island, NY, USA) supplemented with 10% fetal bovine serum (Hyclone, Logan, UT, USA). Cells were transfected using the calcium phosphate method as described previously [14].

### 3.2. Recombinant Plasmids

Construction of the pcDNA4c-MCPyV LT and pcDNA4c-MCPyV Ori plasmids have been described previously [14]. For the phospho-mutant constructs T271A, T297A and T299A, synthetic oligonucleotides containing the desired mutation were annealed with denatured template plasmid pcDNA4c-MCPyV LT and extended with *Pfu Turbo* polymerase (Agilent Technologies, Santa Clara, CA, USA) by QuikChange PCR following the manufacturer’s instructions. Unmutated plasmid DNA templates were removed by DpnI digestion, and the remaining DNA was used to transform DH5α competent cells. To generate IgG-IgG-TEV (IIT) affinity-tagged constructs, a DNA sequence encoding two IgG binding domains of *Staphylococcus aureus* protein A and a TEV protease cleavage site was fused in frame to the N terminus of MCPyV LT constructs using the KpnI site. All constructs were confirmed by DNA sequencing.

### 3.3. Antibodies

The following antibodies were used for immunoflourescent staining: mouse anti-Xpress (R910-25, Invitrogen), rabbit anti-RFC1 (H-300, Santa Cruz, Dallas, TX, USA), rabbit anti-RPA70 (2267, Cell Signaling, Mölndal, Sweden), Alexa Fluor 594 goat anti-rabbit IgG (A11012, Invitrogen), and Alexa Fluor 488 goat anti-mouse IgG (A11001, Invitrogen). The polyclonal rabbit anti-Brd4CA recognizes aa 1313–1362 [14]. Antibodies used for western blotting include: mouse anti-MCPyV LT (CM2B4, Santa Cruz), mouse anti-glyceraldehyde 3-phosphate-dehydrogenase (GAPDH) (G8140-01, US Biological, Salem, MA, USA) and HRP-conjugated horse anti-mouse IgG (7076, Cell Signaling, Danvers, MA, USA).

### 3.4. Sample Preparation for Mass Spectrometry Analysis

HEK 293 cells were transfected with IIT-tagged pcDNA4c-MCPyV LT using the calcium phosphate method. Forty-eight hours post-transfection, nuclear extracts were prepared. Briefly, cells were resuspended in Buffer A supplemented with phosphatase inhibitors (10 mM HEPES [pH 7.9], 10 mM KCl, 0.1 mM EDTA, 30 mM NaF, 1 mM Na_3_VO_4_, 40 mM β-glycerophosphate, 0.2 mM PMSF and protease inhibitors). Cells were swollen on ice for 10 min before NP-40 was added to a final concentration of 6% and then vortexed for 10 s. Nuclei were pelleted by centrifugation at 1200× *g* at 4 °C for 5 min. Nuclei were resuspended in Buffer B supplemented with phosphatase inhibitors (20 mM HEPES [pH 7.9], 400 mM NaCl, 1 mM EDTA, 30 mM NaF, 1 mM Na_3_VO_4_, 40 mM β-glycerophosphate, 0.2 mM PMSF and protease inhibitors) and lysed by passing through a 22-gauge needle 10 times. Lysates were incubuated at 4 °C for 1 h and clarified at 20,000× *g* at 4 °C for 15 min. The supernatant was then immunopurified with IgG-Sepharose 6 Fast Flow (GE Healthcare, Pittsburgh, PA, USA) beads (pre-blocked with 1% BSA) for 2 h at 4 °C. Bound immune complexes were washed twice with IP wash buffer with phosphatase inhibitors (10 mM Tris-HCl [pH 8.0], 150 mM NaCl, 0.1% NP-40, 30 mM NaF, 1 mM Na3VO4, and 40 mM β-glycerophosphate), once with wash buffer without phosphatase inhibitors, and finally once with cleavage buffer (10 mM Tris-HCl [pH 8.0], 150 mM NaCl, 0.1% NP-40, 0.5 mM EDTA, 0.5 mM EDTA, and 1 mM DTT). Bound proteins were then cleaved with TEV protease (Invitrogen) in cleavage buffer for 2 h at room temperature.

Beads were spun down and the supernatant was boiled in sample buffer and resolved by SDS-PAGE. The gel was stained with Coomassie Brilliant blue. The band corresponding to MCPyV LT was excised and analyzed by mass spectrometry.

### 3.5. Mass Spectrometry Analysis

The mass spectrometry analysis was provided by the Proteomics Core Facility, University of Pennsylvania. Protein samples were digested with trypsin as described by Strader *et al.* [34]. Digested peptides were then purified by liquid chromatography using standard purification techniques or with a titanium oxide column (GE Healthcare Biosciences, Pittsburgh, PA, USA). Peptides were analyzed by nanoLC/MS/MS with a LTQ Mass Spectrometer (Thermo Scientific, Waltham, MA, USA) equipped with a nano LC-2D HPLC system (Eksigent, Framingham, MA, USA). The data were analyzed with Sequest and Scaffold software.

### 3.6. Sequence Alignment of Polyomavirus LT Proteins

Clone Manager 9 software was used to align the amino acid sequences of various polyomavirus LT proteins. Amino acid sequences were aligned using the Mult-Way view and BLOSUM 62 scoring matrix. Polyomavirus LT sequences and their NCBI accession numbers are as follows: Merkel Cell Polyomavirus LT, R17a isolate (HM011555.1); Murine Polyomavirus LT (NC_001515.1); African Green Monkey Polyomavirus LT (NC_004763.2); WU Polyomavirus LT (NC_009539.1); KI Polyomavirus LT (NC_009238.1); BK Polyomavirus LT (NC_001538.1); JC Polyomavirus LT (NC_001699.1); SV40 LT (NC_001669.1).

### 3.7. Phyre2 Modeling of MCPyV LT Fragments

We modeled MCPyV LT amino acids 290–433 using Phyre2 software’s intensive mode [35]. The PDB file was then visualized using PyMOL software [36]. The model was oriented using the origin binding domain crystal structure reported by Harrison and colleagues as a reference [30].

### 3.8. Immunoflourescent (IF) Staining

C33A cells were fixed with 3% paraformaldehyde in PBS for 20 min at 4 °C. Cells were incubated in blocking/permeabilization buffer (0.5% Triton X-100 and 3% BSA in PBS) for 10 min at room temperature and stained with specific primary antibodies (as described in the legends) at room temperature for 60 min. After incubation, the cells were washed three times with blocking/permeabilization buffer and incubated with Alexa Fluor 594 goat anti-rabbit IgG and 488 goat anti-mouse IgG (Invitrogen, Molecular Probes, Ashburn, VA, USA) for an additional 60 min. After incubation with the secondary antibodies, cells were counterstained with DAPI (4',6'-diamidino-2-phenylindol) and examined with an Olympus IX81 inverted fluorescence microscope.

### 3.9. Microscopy and Image Analysis

All immunofluorescent images were collected using an inverted fluorescence microscope (Olympus IX81) connected to a high-resolution charge-coupled device camera (QImaging, FAST1394). Images were analyzed and presented using SlideBook 5.0 software (Intelligent Imaging Innovations, Inc., Denver, CO, USA). The scale bars were added using ImageJ software.

### 3.10. Southern Blotting

Replication assays were performed as described previously [13]. Briefly, MCPyV LT constructs were transfected into C33A cells using the calcium phosphate method. Forty-eight hours post-transfection, whole genomic DNA was extracted. 15 µg total DNA was digested with BamHI, treated with or without DpnI at 37 °C overnight, and separated on a 0.7% agarose gel. DNA was transferred to a Hybond-N+ nitrocellulose membrane (Amersham, Piscataway, NJ, USA) and hybridized with a pcDNA4c-MCPyV Ori probe labeled with [α-^32^P] dCTP using Prime-It II random primer labeling kit (Agilent Technologies) per the manufacturer’s instructions. The results were analyzed using a Phosphorimager (Typhoon 9400; GE Healthcare).

### 3.11. Western Blotting

Cells were lysed in hypertonic lysis buffer (10 mM HEPES [pH 7.9], 500 mM NaCl, 3 mM MgCl_2_, 1 mM dithiothreitol, 1 mM phenylmethylsulfonyl fluoride, and protease inhibitors) by passage through a 22-gauge needle 10 times. After a 20-min incubation on ice, the soluble and insoluble fractions were separated by centrifugation at 5,000 rpm for 10 min at 4 °C. The supernatants (20 μg) were resolved on an SDS-PAGE gel and transferred to PVDF membrane. Membranes were blocked in 5% PBST-milk for 1 h at room temperature and incubated in PBST-milk containing primary antibodies at room temperature for 1 h. After washing three times with PBST, membranes were then incubated with HRP-conjugated secondary antibodies in PBST-milk for 1 h at room temperature. Western blots were developed using ECL solution and images were captured using a Fuji imaging system.

### 3.12. Affinity Purification of MCPyV LT

HEK 293 cells were transfected with constructs expressing IIT-tagged MCPyV LT (wild-type or mutant) using the calcium phosphate method. Forty-eight hours post-transfection, nuclear extracts were prepared. Briefly, cells were resuspended in Buffer A (10 mM HEPES [pH 7.9], 10 mM KCl, 0.1 mM EDTA, 0.2 mM PMSF and protease inhibitors). Cells were swollen on ice for 10 min before NP-40 was added to a final concentration of 6% and then vortexed for 10 s. Nuclei were pelleted by centrifugation at 1200× *g* at 4 °C for 5 min. Nuclei were resuspended in Buffer B (20 mM HEPE [pH 7.9], 400 mM NaCl, 20% glycerol, 1 mM EDTA, 0.2mM PMSF and protease inhibitors) and lysed by passing through a 22-gauge needle 10 times. Lysates were clarified at 20,000× *g* at 4 °C for 15 min. The supernatant was then immunopurified with IgG-Sepharose 6 Fast Flow (GE Healthcare) beads pre-blocked with 1% BSA for 2 h at 4 °C. Bound immune complexes were washed three times with IP 150 buffer (10 mM Tris-HCl [pH 8.0], 150 mM NaCl, 0.1% NP-40) and once with TEV cleavage buffer (50 mM Tris-HCl [pH 8.0], 6% glycerol, 0.5 mM EDTA, 0.5 mM DTT, 1 mM PMSF). Bound proteins were then cleaved with TEV protease (Invitrogen) in TEV cleavage buffer, overnight at 4 °C. The beads were spun down and the supernatant collected for further biochemical analysis.

### 3.13. Electromobility Shift Assays (EMSA)

The EMSA probe was generated by PCR amplifying a portion of pcDNA4c-MCPyV Ori (Forward Primer: TTG GCA GAG GCT TGG GGC TCC, Reverse Primer: GCG GAA TTC TAA GCC TCT TAA GCC TC). The PCR product was purified using a Qiagen PCR Purification Kit (Cat# 28104) following the manufacturer’s instructions. The purified probe (100 ng) was then 5' labeled with [^32^P-γ] ATP with T4 Polynucleotide Kinase (New England Biolabs, Ipswich, MA, USA) following the manufacturer’s instructions. The labeled probe was then diluted 1:50 before being used in the EMSA.

Binding reactions (20 µL) were assembled on ice. Various amounts of affinity purified MCPyV LT was mixed with 40 fmol labeled probe in binding buffer (30 mM Tris-HCl [pH 8.0], 10% glycerol, 0.5 µg BSA, 10 ng poly d(I-C), 40 ng Sonicated Salmon Sperm DNA, 5 mM AMP-PNP, 1 mM DTT, and 1 mM PMSF). Reactions were incubated at room temperature for 25 min and then separated on a 4% non-denaturing polyacrylamide gel in 0.5 × TBE at 120 V for 3 h. The gel was dried and subjected to autoradiography. The non-hydrolyzable ATP analogue, adenylyl imidodiphosphate (AMP-PNP) was obtained from Sigma (St. Louis, MO, USA, Cat# A2647).

### 3.14. Unwinding Assay

The probe was generated by annealing two oligos (80 ng each) spanning the central portion of the MCPyV Ori sequence (Figure 6A) (Top Strand Oligo: GTG ACT TTT TTT TTT CAA GTT GGC AGA GGC TTG GGG CTC CTA GCC TCC GAG GCC TCT GGA AAA AAA AGA GAG AGG CC; Bottom Strand Oligo: CAG AGG CCT CTC TCT TTT TTT TCC AGA GGC CTC GGA GGC TAG GAG CCC CAA GCC TCT GCC AAC TTG AAA AAA AAA AGT CAC). The four-nucleotide overhang was filled in using Klenow DNA Polymerase (New England Biolabs) by mixing the duplexed probe in a reaction containing 0.1 mM dTTP, dGTP, and [^32^P-α] dCTP and incubating at room temperature for 20 min. After adding dATP to a final concentration of 0.1mM, reactions were incubated for another 20 min at room temperature.

Various amounts of purified MCPyV LT were combined with 60 pg of labeled probe in 20 µL unwinding reaction buffer (30 mM HEPES [pH 8.0], 0.1 mg/mL BSA, 7 mM MgCl2, 4 mM ATP, 40 mM creatine phosphate, 25 µg/mL creatine phosphate kinase). Reactions were carried out at room temperature for 1 h. Reactions were stopped by adding 5 µL 5 × Stop Buffer (2.5% SDS, and 100 mM EDTA). Loading dye (bromophenol blue, 4% sucrose and 1XTBE) was added and samples were separated on an 11% non-denaturing PAGE in 0.5XTBE. Gels were dried and exposed for autoradiography.

### 3.15. Helicase Assay

The helicase assay was performed as previously described with minor modification [12]. Wild-type or mutant MCPyV LT fused to an IIT tag was expressed in HEK 293 cells and purified using IgG Sepharose 6 Fast Flow (GE Healthcare), which were preblocked with 1% BSA in PBS at 4 °C for >1 h. Beads with bound LT were split into two equal fractions for SDS-PAGE/Coomassie brilliant blue staining and helicase assays, respectively. To label the helicase assay substrate, 35 ng of a 31-mer oligo (5'-CCA GGG TTT TCC CAG TCA CGA CGT TGT AAA C-3') was annealed to 1 μg of M13mp18 DNA (New England BioLabs). The primer was then elongated using Klenow polymerase (New England BioLabs) in a 50 μL reaction containing 0.1 mM dCTP, dGTP, and [α-^32^P] dATP. After 20 min of incubation at room temperature, 0.1 mM dATP was added for 20 min. Then, 0.5 μL of labeled substrate was used in each reaction. LT purified on IgG Sepharose was incubated with the substrate in helicase assay buffer (20 mM Tris-HCl [pH 7.5], 10 mM MgCl_2_, 1 mM DTT, 0.1 mg/mL BSA, and 5 mM ATP) at 37 °C for 30 min. The reaction was stopped by adding SDS to a final concentration of 0.2% and EDTA to 50 mM. Total reaction mixtures were resolved by electrophoresis on 11% non-denaturing polyacrylamide gels. The gels were dried and subjected to autoradiography.

### 3.16. Statistical Analyses

Prism software was used to perform a one-way ANOVA test. A *p* < 0.05 was considered statistically significant.

## 4. Discussion

Merkel Cell Polyomavirus is the first human polyomavirus linked to a human cancer. As such, it has garnered a considerable amount of interest, especially with regards to its oncogenic potential and its causative role in MCC. Much of the basic virology of MCPyV, in contrast, has been lacking, in large part due to the difficulty in propagating the virus and the lack of a natural host cell line. Previous work from our lab has established that MCPyV LT interacts with the host DNA damage response machinery, potentially to regulate viral genome replication [13]. Understanding how polyomavirus replication is regulated will be critical for understanding the very early steps of MCPyV-induced transformation and oncogenesis.

Phosphorylation has been a well-established mechanism of regulation for SV40 LT activities, especially for genome replication. In addition, MCPyV LT has a unique stretch of amino acids that is rich in serines and threonines, offering many new potential phosphorylation sites and therefore mechanisms of regulation. To search for relevant sites in a relatively unbiased fashion, we performed a proteomic analysis of ectopically expressed MCPyV LT (Figure 1 and Supplementary Table S1). This analysis identified three threonines that are likely phosphorylated when MCPyV LT is expressed: T271, T297 and T299. We generated alanine substitutions of these sites to probe their function.

T271 was immediately interesting to us for a variety of reasons. It was independently identified in multiple peptides in both standard and titanium oxide purifications (Supplementary Table S1), providing us with a high degree of confidence that this site is phosphorylated in cells. More intriguing, this site is located in the unique region of MCPyV LT (aa 95–290). It does not have any homologies to other polyomavirus LT proteins analyzed (Figure 1B). We anticipate phosphorylation at this site may represent a novel function that MCPyV has acquired. Efforts thus far, however, have not revealed what those functions may be. The T271A mutant’s ability to bind Brd4 and activate the host DDR is similar to that seen for WT LT (data not shown). Additionally, this mutant was able to form replication foci and replicate plasmids containing the Ori almost as well as WT (Figure 3 and Figure 4). Additional experiments will be performed to identify its role in MCPyV infection.

T299, in contrast, is highly conserved among all polyomavirus LT proteins analyzed (Figure 1B). This site is homologous to T124 in SV40 LT, which has been extensively studied for its role in regulating LT-mediated DNA replication. Alanine substitution of this site in SV40 LT completely abrogated replication. Biochemical analysis of T124A mutants showed that it had somewhat impaired double-hexamer interactions and unwinding activity [23,24]. More importantly, its ability to unwind duplex Ori DNA was abrogated while basic helicase activity remained unperturbed [24]. In line with these findings, T299A in MCPyV LT also failed to replicate plasmids containing the viral Ori (Figure 4) and did not form replication foci (Figure 3). This mutant had a reduced capacity to bind the viral Ori in EMSA experiments (Figure 5). Although studies of the homologous LT mutant, T124A, in SV0 demonstrated that unwinding of the origin was attenuated, we were unable to reproduce this finding in our studies [20]. While it is possible that MCPyV LT behaves differently from other polyomavirus LT’s, we believe technical limitations in our hands are more likely responsible for not seeing this phenotype in MCPyV T299A LT. Its helicase activity remained identical to wild-type (Figure 6D), which has been reported for T124A LT in SV40 [20]. Our EMSA studies did not indicate an attenuated double-hexamer phenotype as shown by Barbaro and colleagues for SV40 LT [23], and the unwinding phenotype we observe is extremely subtle. It is possible our experimental conditions are not conducive for revealing these phenotypes, or (less likely) that T299 phosphorylation behaves in a slightly different biochemical manner than T124 in SV40 LT. We conclude that phosphorylation of T299 is required for MCPyV LT to initiate replication of its genome in ways similar to T124 phosphorylation in SV40.

T297 was not well conserved among the polyomavirus LT proteins analyzed. Modeling of this site seemed to indicate that this residue might face and even interact with DNA when the LT OBD engages the viral genome (Figure 2A). We speculated this site might have an impact on DNA replication. Indeed, the T297A mutant had twice as many LT-positive nuclei exhibiting replication foci as WT LT (Figure 3D). Supporting this observation, Southern blotting of *in cellulo* replication products showed that this mutant replicated plasmids containing the viral origin to a very high degree (Figure 4). Biochemical analyses revealed a strikingly robust affinity for the viral Ori (Figure 5), while unwinding and helicase activities remained largely unaffected (Figure 6). These data indicate that phosphorylation of this site would dramatically decrease LT’s capacity to bind the viral Ori, which would presumably limit its ability to initiate viral replication. These observations are in line with our structural model (Figure 2) predicting that this site faces the OBD/DNA binding interface. The negative charge of a phosphate moiety at this site would presumably clash with the negatively charged phosphate-backbone of DNA, leading to reduced DNA binding. SV40 has also been reported to have phosphorylation sites that negatively impact replication. Phosphorylation at serines 120 and 123 was shown to have a negative effect on replication [27]. Threonine 297 may provide a similar regulatory function for MCPyV LT. It is possible that phosphorylation at a site neighboring the stimulatory threonine (124 for SV40 LT, 299 for MCPyV LT) may be a general feature of polyomavirus LT proteins to limit Ori recognition and to provide a brake for viral replication.

We attempted to generate phosphomimetic mutants (threonine to aspartate or glutamate) of these sites to probe these dynamics more closely; however, these mutants behaved just like alanine substitutions (data not shown). Interestingly, a MCPyV LT expression construct containing both T297A and T299A mutations matched the T299A phenotype completely: it failed to form replication foci or replicate plasmids with viral Ori’s (data not shown). The T297A mutation would allow for enhanced binding of the origin (Figure 5), but the T299A mutation, which likely acts at steps after Ori binding during initiation of replication, completely abrogated replication of this double mutant (data not shown).

Taken together, our data support a model where T299 and T297 phosphorylation act as antagonistic ON and OFF switches for replication, respectively. We would hypothesize that T299 is first phosphorylated to stimulate viral replication, while subsequent phosphorylation at threonine 297 would abrogate Ori recognition and presumably reduce viral genome replication, possibly in favor of late gene expression and/or packaging. Phosphatases may also play a role, either removing phosphates from T299 to halt replication or from T297 to allow replication to continue. Without antibodies specific for phosphorylation at these sites, it is difficult to track when these sites become phosphorylated during infection or to begin searching for the kinases that add these marks during infection. Analysis of the amino acid sequences of these sites offer some clues. For SV40, cdc2/CDK1 was shown *in vitro* to be responsible for phosphorylation at T124, the homologue to MCPyV LT T299 [19]. The residues surrounding both MCPyV LT T299 and SV40 LT T124 (TPPK for both viruses, see Figure 1B) exhibit a classic cdc2/CDK1 consensus sequence (S-P-X-basic residue) [37]. Although it was not directly tested here, it is likely that cdc2/CDK1 plays a role in phosphorylating T299 during MCPyV infection. Casein kinase II was also shown to phosphorylate nearby serine residues in SV40 LT *in vitro*, which played a role in SV40 LT nuclear import [38,39,40,41]. Finally, ATM kinase has been shown to phosphorylate SV40 LT in this region as well, contributing to LT-mediated replication [42]. The threnonines T271 and T297 identified in this study do not exhibit homologies to the known consensus sequence of either of these kinases, indicating that other kinases are likely involved. Finally, other MCPyV viral proteins, like sT antigen, 57 kT antigen and ALTO [43], may affect when, where and how LT is phosphorylated during the viral life cycle. These questions should be explored further as more reagents and cell lines become available for MCPyV studies.

One of the hallmark features of MCPyV LT in MCC is that the protein frequently becomes mutated such that it is expressed in a truncated fashion [4]. These truncations almost always delete the helicase domain and the OBD. Interestingly, the three phosphorylation sites identified in this study are almost always omitted from the truncated proteins as well. It has been hypothesized that LT becomes truncated to avoid replicating the integrated viral genome, which would presumably cause genomic instability [1]. In line with this reasoning, at least one MCPyV related MCC tumor has been identified with a full-length LT protein but the integrated viral genome contains a mutated Ori that fails to support viral replication [4,31]. Given that mutation of T299 completely abolishes LT’s capacity to replicate the viral origin, it is interesting to note that this mutation has never been observed in any of the MCC cases studied thus far. It is possible that the OBD, helicase domain and/or extreme C-terminal domain contain additional activities beyond replication that are negatively selected out during MCC progression. Our previous studies have indicated that the C-terminal half of MCPyV LT interacts with the p53 pathway to maintain cells in a stalled S-phase, which may be conducive to viral genome replication but antithetical to tumorigenesis [12]. Others have similarly postulated that the C-terminal domain contains activities beyond replication that are negatively selected during MCC oncogenesis [44]. Further investigation of this region of the protein may provide a broader and more comprehensive understanding of how MCPyV LT manipulates the host cell, and how these activities become disrupted during MCC tumorigenesis.

## 5. Conclusions

Our work identifies three phosphorylation sites on MCPyV LT; T271, T297 and T299. T299 is conserved among all polyomavirus LT proteins analyzed, and when phosphorylated stimulates viral replication, as has been reported for SV40 LT. T297 is a novel phosphorylation site that may negatively regulate viral replication by reducing the binding affinity of LT for the viral origin. T271 did not have an effect on viral replication, but was highly enriched in the mass spectrometry analysis and is located in a unique region of MCPyV LT; further investigation is required to uncover what novel functions this site may have during the viral life cycle.

## Supplementary Files

Supplementary File 1
