# Supplementary material for: Phosphorylation of Large T Antigen Regulates Merkel Cell Polyomavirus Replication
Source: Cancers (Basel). 2014 Jul 8;6(3):1464–86. doi: 10.3390/cancers6031464 (PMC4190550; doi:10.3390/cancers6031464)
Supplement: Supplementary File 1 [file cancers-06-01464-s001.pdf]

Supplementary Material

Table S1. Phosphorylated peptides Identified by mass spectrometry. Shown are the reconstructed peptides isolated by both the standard purification and titanium oxide enrichments after trypsin digest. Confidence in identification is given as a probability. The residues in bold are the ones identified as having a mass value 80 daltons higher than predicted, indicating a phosphorylation modification. Please fill data into the table format below?

Peptide Purification	Peptide Sequence	Probability	Amino Acid	Modification
Standard	(R)SSASSASSASF**T**STPPKPK(K)	95%	Thr 297	Phospho (+80)
	(R)SSSFTTPK**T**PPPFSR(K)	95%	Thr 271	Phospho (+80)
	(R)SSSFTTPK**T**PPPFSR(K)	95%	Thr 271	Phospho (+80)
Titanium Oxide	(R)SSASSASSASFTS**T**PPKPK(K)	95%	Thr 299	Phospho (+80)
	(R)SSASSASSASFTS**T**PPKPK(K)	95%	Thr 299	Phospho (+80)
	(R)SSSFTTPK**T**PPPFSR(K)	95%	Thr 271	Phospho (+80)
	(R)SSSFTTPK**T**PPPFSR(K)	95%	Thr 271	Phospho (+80)
	(R)SSSFTTPK**T**PPPFSR(K)	95%	Thr 271	Phospho (+80)

Figure S1. MCPyV phospho-mutant LT proteins recruit cellular factors to the same degree. Replication foci scored in Figure 3 A-C were assessed for colocalization of the indicated cellular factors. Bar indicates standard deviation from the mean from three independent experiments. A one-way ANOVA was performed to determine significance (** $p < 0.001$).

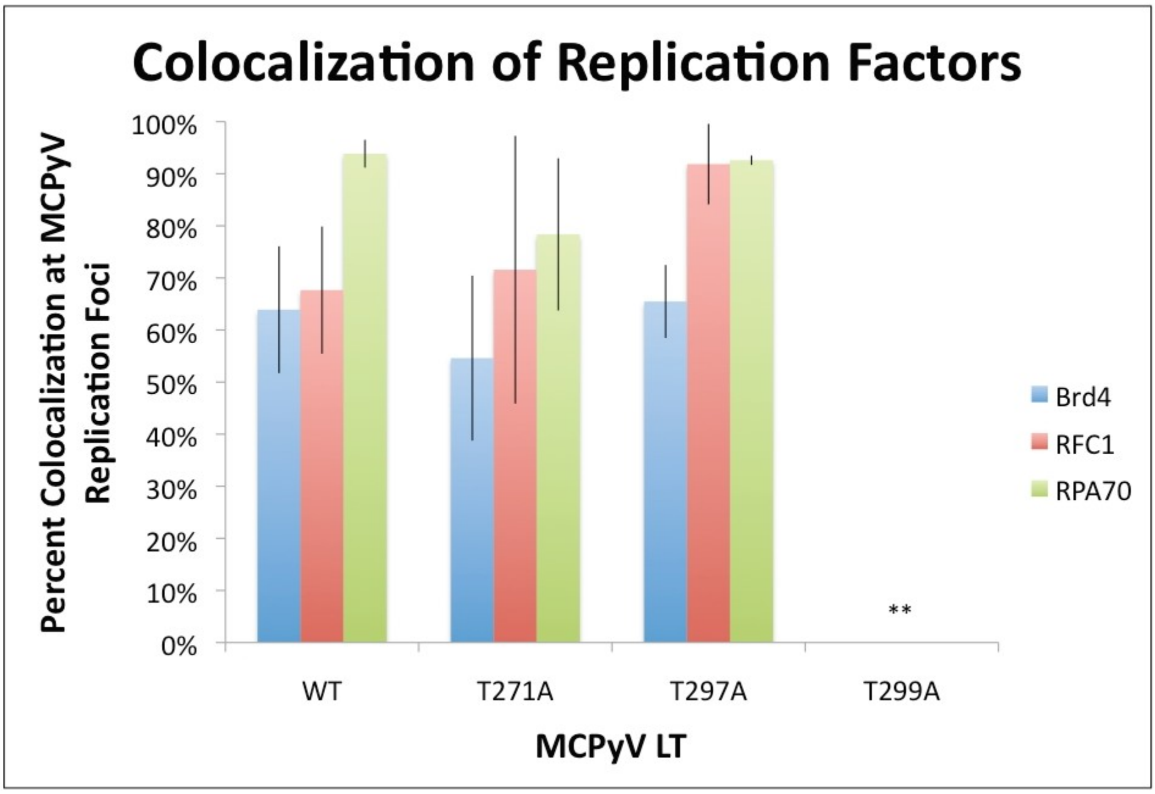

Figure S2. Unwinding assay performed with a helicase mutant LT and a mutated Ori probe. Unwinding assays were performed with both wild-type LT and the E627A mutation, which abolishes helicase activity. In parallel, an origin probe with a mutation in the seventh pentanucleotide repeat which abolishes origin-dependent replication (Ori350) was also tested using wild-type MCPyV LT. Data shown are representative of two repeats.

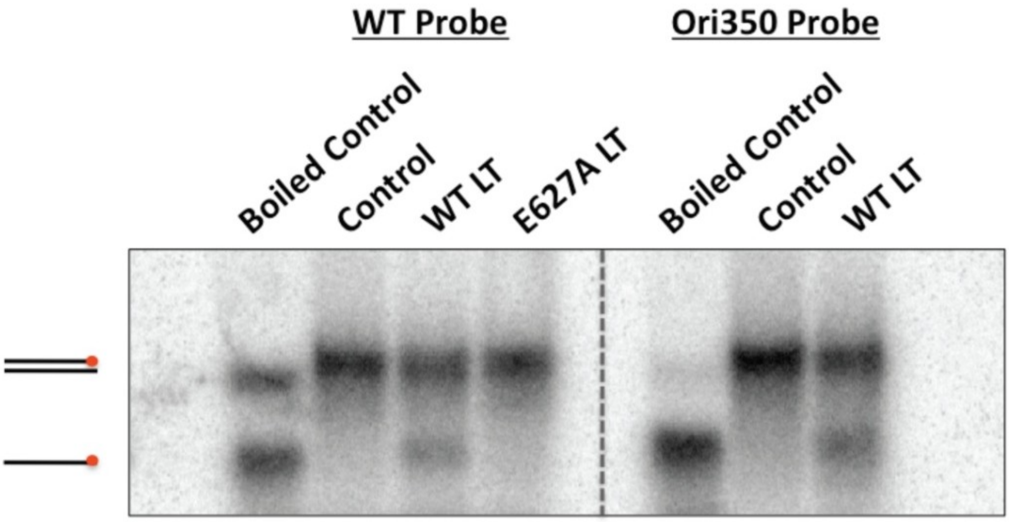

© 2014 by the authors; licensee MDPI, Basel, Switzerland. This article is an open access article distributed under the terms and conditions of the Creative Commons Attribution license (http://creativecommons.org/licenses/by/3.0/).
